# Associations between left ventricular structure and function with cardiorespiratory fitness and body composition in individuals with cervical and upper thoracic spinal cord injury

**DOI:** 10.1038/s41393-020-00591-4

**Published:** 2020-12-07

**Authors:** Abdullah A. Alrashidi, Shane J. T. Balthazaar, Katharine D. Currie, Tom E. Nightingale, Andrei V. Krassioukov

**Affiliations:** 1 International Collaboration On Repair Discoveries, University of British Columbia, Vancouver, BC Canada; 2 Department of Experimental Medicine, University of British Columbia, Vancouver, BC Canada; 3 Department of Physical Therapy, King Fahad Medical City, Riyadh, Saudi Arabia; 4 Department of Kinesiology, Michigan State University, East Lansing, MI USA; 5 School of Sport, Exercise and Rehabilitation Sciences, University of Birmingham, Edgbaston, Birmingham, UK; 6 Division of Physical Medicine and Rehabilitation, University of British Columbia, Vancouver, BC Canada; 7 G.F. Strong Rehabilitation Centre, Vancouver, BC Canada

**Keywords:** Spinal cord diseases, Rehabilitation

## Abstract

**Study design:**

Cross-sectional.

**Objective:**

It is known that left ventricular mass (LVM) and cardiorespiratory fitness (CRF) are associated to fat-free mass (FFM).  It is unknown if these factors associated with left ventricular (LV) structure and function outcomes in individuals with spinal cord injury (SCI).

**Setting:**

University-based laboratory.Vancouver, BC, Canada.

**Methods:**

Thirty-two individuals (aged 40 ± 11 years) with chronic, motor-complete SCI between the fourth cervical and sixth thoracic levels were recruited. Echocardiographic LV parameters and body composition were assessed at rest, as per the recommended guidelines for each technique. CRF was assessed during an incremental arm-cycle exercise test until volitional fatigue. The appropriate bivariate correlation coefficients [i.e., Pearson’s (*r*) and Spearman’s rank (*R*_*s*_)] tests were used for normal and non-normal distributed variables, respectively.

**Results:**

LV structure and function parameters were not associated with the indexed peak oxygen consumption (V̇O_2peak_) [i.e., relative to body weight or FFM] (*R*_*s*_ values ranged from −0.168 to 0.134, all *P* values > 0.223). The association between peak oxygen pulse and the resting echocardiographic-obtained SV was medium sized (*R*_*s*_ = 0.331, *P* = 0.069). The LVM associations with FFM and fat mass (FM) were large and small (*r* = 0.614, *P* < 0.001 and *r* = 0.266, *P* = 0.141, respectively). Associations of absolute V̇O_2peak_ were medium- positive with FFM (*R*_*s*_ = 0.414, *P* = 0.021) but negative with FM (*R*_*s*_ = −0.332, *P* = 0.068).

**Conclusion:**

LV parameters measured at rest are not associated with V̇O_2peak_ in individuals with cervical and upper-thoracic SCI. Given the observed associations between LVM and V̇O_2peak_ with FFM, future studies may consider utilizing FFM for indexing cardiovascular measures following SCI.

## Introduction

It is well-established in non-injured individuals that higher levels of cardiorespiratory fitness (CRF) are associated with a reduced risk of cardiovascular disease (CVD), all-cause mortality, and other inactivity-related chronic diseases [1]. Individuals with spinal cord injury (SCI), are known to have a reduced CRF, which is in part due to increased levels of physical inactivity following SCI [2, 3]. Furthermore, individuals with motor-complete, cervical injuries tend to display lower levels of CRF [2, 4]. Collectively, this increases the risk of CVD events and cardiometabolic dysfunction, all of which negatively impact independence and quality of life in this population [5].

In the non-injured population, prolonged inactivity and decreased hemodynamic load, even with the absence of apparent CVD, have been linked with a smaller cardiac cavity size and mass [6]. A recent meta-analysis revealed a reduction in left ventricular mass (LVM), reduced stroke volume (SV), and altered diastolic function in individuals with SCI compared to the non-injured population [7]. Alongside reduced CRF and lower hemodynamic load, injuries at and above the sixth thoracic (≥T6) spinal segment not only diminishes sympathetic control to the peripheral vasculature but may also compromise sympathetic drive to the heart (extends from between the T1–T5 spinal segments) [8]. Left ventricular (LV)  dysfunction and perturbed autonomic function [9] may limit cardiac output (Q̇) and SV, in turn reducing the maximal attainable level of CRF [i.e., lower peak oxygen consumption (V̇O_2peak_)]. CVD events are elevated in individuals with SCI [10]; therefore, assessing LV dysfunction in conjunction with reduced CRF is highly relevant, given studies have shown that both are linked with increased risks of CVD morbidity and mortality in non-injured and select clinical populations [1, 11, 12].

The association between resting echocardiographic LV parameters and CRF has been investigated in non-injured individuals and certain clinical populations. The results of these studies were controversial with some reporting there is an association [13–15] while other reported that this association does not exist [16, 17]. The association between CRF and LV diastolic function is more evident compared to that with systolic function [14, 15, 18]. One mechanism that may explain the association between LV diastolic function and CRF is the capability of LV to generate maximal Q̇ via the maintenance of adequate LV filling pressure [13].

Cardiac dimensions are very closely associated with body dimensions (e.g., body mass and height). Thus, for the accurate quantification of cardiac dimensions and functional measures, as well as for inter- and intra-group comparison, the effect of body size should be partitioned out. Previous studies involving non-injured individuals have shown that LVM is closely related to body size and composition, as well as demonstrating that fat-free mass (FFM) is strongly associated with and a reliable predictor of LVM compared with other body dimensions [e.g., body surface area (BSA), body mass, height] [19, 20]. While it is common to report LVM indexed to BSA [21], such indexing might underestimate LVM measurements and fail to identify individuals with obesity and LVM abnormalities [22]. Individuals with SCI, as a result of paralysis, are at increased risk of obesity and altered body composition compared to non-injured individuals [23]. Thus, indexing LVM to BSA may pose the same problem with this population.

The purpose of this study was to investigate the associations between LV structure and function with CRF. We hypothesized that those with a higher V̇O_2peak_ would have more favorable resting LV parameters. Due to the observed association between LVM and body composition in the non-injured population, we also explored this association in our cohort (i.e., cervical and upper-thoracic SCI).

## Methods

### Participants

These data were collected from a larger prospective, multicenter, randomized clinical trial [The Cardiovascular Health/Outcomes: Improvements Created by Exercise and Education in SCI (CHOICES)] conducted at the University of British Columbia, with the registration number (NCT01718977) on ClinicalTrial.gov, November 1, 2012, the study protocol of which has been previously published [24]. All participants provided informed consent and all measurements were collected as part of the baseline assessments of CHOICES (between April 2013 and August 2017) before the commencement of the training interventions. Leisure time physical activity was captured using the recently validated Leisure Time Physical Activity Questionnaire for People with SCI (LTPAQ-SCI) [25]. All participants arrived at the laboratory following a minimum 4 h fast and avoided vigorous physical activity (PA) for the preceding 24 h. This study included the data of participants who were recruited from the International Collaboration On Repair Discoveries (ICORD), Vancouver, BC, Canada, in which echocardiography was performed. The inclusion criteria were as follows: Adults 18–60 years of age with chronic [time since injury (TSI) > 1 year], motor-complete [American Spinal Injury Association Impairment Scale (AIS) A–B)], traumatic SCI between the fourth cervical (C4) and T6 segments. The neurological level of injury (NLI) and AIS were assessed and determined using the International Standards for Neurological Classification of SCI [26]. Exclusion criteria included any medical history or symptoms of CVD, major trauma/surgery in the last 6 months, an unstable medical/psychological condition, or any cognitive dysfunction that would be a barrier to understanding English.

### Outcome measures

#### LV structure and function

All participants underwent non-invasive trans-thoracic echocardiography (Vivid 7, GE Healthcare, Horton, Norway) in the left lateral decubitus position, as per the recommendations of the American Society for Echocardiography [27]. Images were collected and analyzed offline via dedicated software (EchoPAC; GE Healthcare, Horton, Norway). The average of three cardiac cycles was used to determine LV structure and global functions. Measures of LV structure at the end-diastole and end-systole were reported from the parasternal long-axis views. LVM was calculated and indexed to BSA according to a well-established formula [28] (i.e., the Devereux method). Volumetric measurements and systolic functions were derived from the apical four-chamber biplane views using the modified Simpson method. Q̇ was calculated as the product of SV and heart rate (HR). The LV diastolic function comprised early peak mitral annular septal tissue velocity (E’); early (E) and late (A) peak transmitral flow, and the early-to-late transmitral filling velocity (E/A) ratio, which were analyzed using the pulsed-wave Doppler. The E/E’ ratio, a validated estimate of LV filling pressure, was calculated. Additional diastolic indices included deceleration time, defined as the maximum downward slope of the early peak flow rate, and isovolumetric relaxation time, defined as the time interval from the aortic valve closing to the mitral valve opening, as determined on the spectral Doppler trace [27]. Intraclass correlation coefficients (ICCs) were performed on ten randomly selected participants from our cohort, calculating all echocardiography outcomes across three cardiac cycles. ICCs showed a high degree of reliability (mean range: 0.918 to 0.993) with 95% confidence intervals of 0.218–0.998.

#### Cardiorespiratory fitness

An incremental arm-crank exercise test on an electrically braked arm-crank ergometer (Lode BV, Groningen, The Netherlands), done until volitional exhaustion was performed to determine the peak parameters. HR was recorded continuously using a chest-strap HR monitor (T31; Polar Electro Inc., Woodbury, NY, USA). Respiratory gases were collected using a metabolic cart (Parvomedics Truemax 2400, Sandy, UT, USA) calibrated immediately before each test. Participants performed the test while sitting in their wheelchair and were asked to empty their bladder prior to the test to avoid the possible development of autonomic dysreflexia. After two minutes of resting, the test protocol began with a warm-up with no resistance for two minutes and then continued with one-minute stages, where resistance was increased by 5–10 Watts per stage for participants with cervical and upper-thoracic NLI, respectively [29]. The participants were instructed to maintain a cadence of 50 revolutions per minute (rpm) throughout the test. The test continued with verbal encouragement until volitional exhaustion was experienced or the cadence dropped below 30 rpm. The test ended with a two-minute cool-down period with no resistance. Peak gas exchange values were collected, and the highest 20 s average was taken as the peak value. V̇O_2peak_ was normalized to body mass and FFM. Peak oxygen pulse (O_2pulse_) was calculated as a ratio between V̇O_2peak_ and HR_peak_.

#### Body composition

Body composition was measured using Dual-energy X-ray absorptiometry (DXA) (Hologic 4500A or W densitometer, Waltham, Massachusetts, USA). Outcomes were reported as per the International Society for Clinical Densitometry guidelines and as detailed previously in the study protocol [24]. The study coordinator assisted the transfer to the DXA plinth through the use of a sliding board, as necessary. An expert DXA technologist checked the participant’s posture while on the plinth and ensured that all limbs were aligned and without any rotation or shifting of the pelvis and/or trunk. For the purpose of this study, we only reported FFM and fat mass (FM) data in our analysis.

### Statistical analyses

All statistical analyses were performed using the Statistical Package for the Social Sciences (SPSS, version 24; IBM Corporation, Armonk, USA) and GraphPad Prism (version 6). All data were visually inspected and analyzed for normality using a Q–Q plot and Shapiro–Wilk test, respectively. To investigate the association between CRF, LV parameters, and body composition, bivariate correlation coefficient [i.e., Pearson’s (*r*) or Spearman’s rank (*R*_*s*_)] tests were used for normal and non-normal distributed variables, respectively. We also ran partial correlation coefficients controlling for pre-determined covariates (i.e., age, NLI, TSI). The NLI was converted into a continuous variable, where C4 = 1, and C5 = 2, and so on until T6 = 11. The magnitude of each correlation was interpreted using the following: small (*r* > 0.1), medium (*r* > 0.3), large (*r* > 0.5), and very large (*r* > 0.7). Univariate (simple) regression (*R*^2^) was used where appropriate (i.e., predicting LVM from FFM).

## Results

**Fig. 1:**
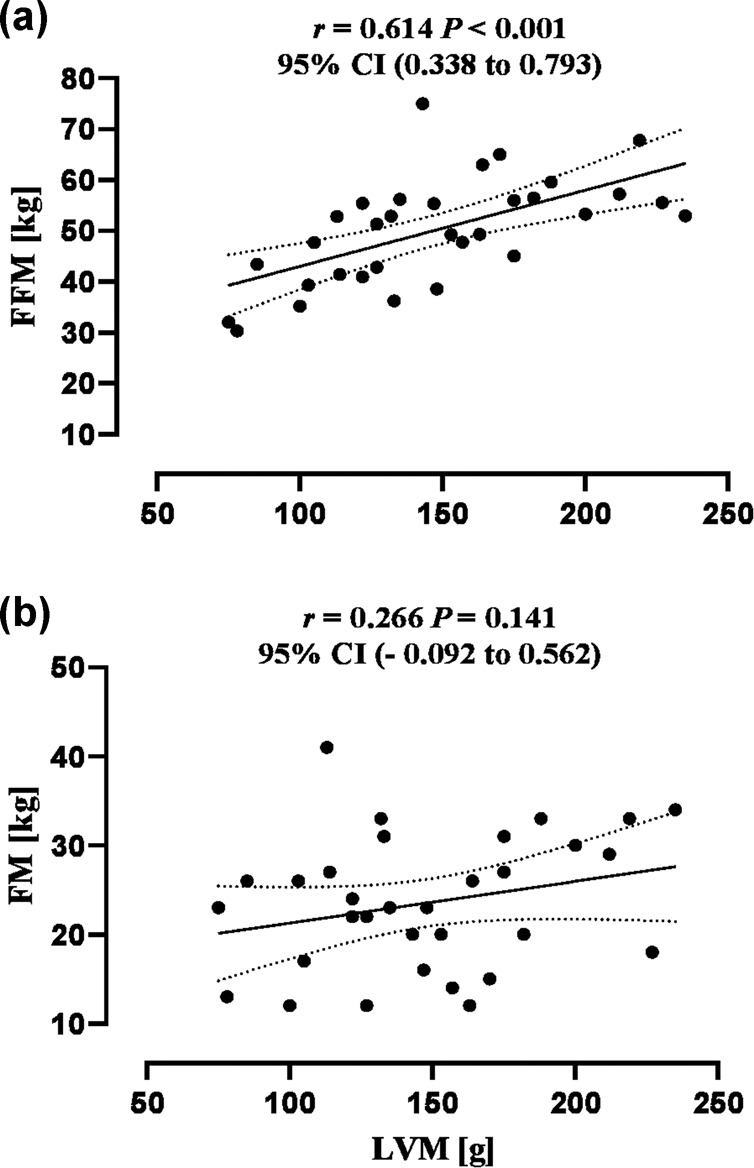
Associations between left ventricular mass (LVM) with fat-free mass (FFM) and fat mass (FM). Association of LVM with FFM (**a**) and FM (**b**). Broken lines denote 95% confidence intervals.

A total of 32 participants were included. Participants’ demographics, injury characteristics, peak CRF, body composition parameters and level of leisure time PA are presented in Table 1. Of the study participants, males and individuals with cervical injury accounted for 75% and 66%, respectively, and the majority were classified as AIS A (72%). None of the resting echocardiographic LV structure and function parameters were associated with relative V̇O_2peak_ (i.e., relative to body mass or FFM) (Table 2). The association between peak oxygen pulse (O_2pulse_), a surrogate of exercise SV that was obtained at peak CRF testing, was medium with resting echocardiographic-obtained SV (*R*_*s*_ = 0.331, *P* = 0.069). Associations between absolute V̇O_2peak_ with FFM were medium- positive (*R*_*s*_ = 0.420, *P* = 0.017) but negative with FM (*R*_*s*_ = −0.360, *P* = 0.043). LVM associations with FFM and FM were large and small (*r* = 0.614, *P* < 0.001 and *r* = 0.266, *P* = 0.141, respectively) (Fig. 1). The association between LVM and FFM remained large after partially controlling for age, NLI, and TSI (*r* > 0.607, *P* < 0.001).

**Table 1:** Participant characteristics.

	Cervical *N* = *21*	Thoracic *N* = *11*
Age (y)		
	41 ± 11	37 ± 10
Sex		
Male/female	15/6	9/2
AIS		
A/B^a^	12/9	11/0
Time since injury (yrs.)	10 (15)	8 (21)
Height (cm)	178 ± 11	176 ± 8
Mass (kg)	76 ± 24	76 ± 14
BMI (kg/m^2^)	23.7 ± 3.3	24.6 ± 3.4
BSA (m^2^)	1.9 ± 0.3	1.9 ± 0.1
Peak cardiorespiratory fitness		
V̇O_2_ (mL/kg/min)^a^	8.17 (3.71)	14.41 (5.69)
V̇O_2_ (mL/kg_FFM_/min)^a^	13.55 (6.14)	19.77 (8.64)
V̇O_2_ (L/min)^a^	0.69 (0.36)	1.04 (0.42)
O_2pulse_ (mL/beat)	6.83 ± 1.97	7.65 ± 2.08
Body composition		
FFM (kg)	48.5 ± 11.8	52.9 ± 7.2
FM (kg)	25.2 ± 7.7	20.6 ± 6.2
FFMI (kg/m^2^)	15.2 ± 2.5	17.2 ± 1.6
Physical activity		
Mild LTPA (min/week)	120 (180)	100 (120)
Moderate LTPA (min/week)	68 (240)	105 (240)
Heavy LTPA (min/week)	35 (90)	70 (270)
Total LTPA (min/week)	300 (505)	315 (627)

Data are mean ± standard deviation for parametric variables (age, height, mass, BMI, BSA, O_2pulse_, FFM, FM and FFMI). Nonparametric variables (time since injury, V̇O_2_ and LTPA) are presented as median (interquartile range). Categorical variables (sex and AIS) are presented as participant numbers.

*AIS* American Spinal Injury Association Impairment Scale, *BMI* body mass index, *BSA* body surface area, *BMI* body mass index, *BSA* body surface area, *V̇O*_*2*_ oxygen consumption, *FFM* fat-free mass, *FM* fat mass, *FFMI* fat-free mass index, *LTPA* leisure time physical activity.

^a^Indicates significant between-group differences at 0.05 (Chi-squared and Mann-Whitney U tests for AIS and ${\dot {\mathrm{{V}}}}{\mathrm{{O}}}_{\mathrm{2peak}}$, respectively).

**Table 2:** Bivariate association between CRF and resting LV parameters.

		LV parameters					
	*Mean* ± *SD (CRF)*	SV (mL)	Q̇ (L/min)	LVM (g)	E/A ratio	E/E’ septal	Deceleration time (ms)
*Mean* ± *SD (LV)*		57.47 ± 9.18	3.59 ± 0.87	147.8 ± 42.7	1.59 ± 0.59	7.12 ± 2.14	221 ± 75
CRF							
V̇O_2peak_ (mL/kg/min)	11.23 ± 4.43	−0.047	0.088	−0.225	0.050	−0.024	−0.076
V̇O_2peak_ (mL/kg_FFM_/min)	17.35 ± 4.43	−0.066	−0.078	−0.168	0.134	−0.046	−0.044

*SV* stroke volume, *Q̇* cardiac output, *LVM* left ventricular mass, *E/A ratio* ratio of early-to-late transmitral.

velocity, *E/E’* mitral peak E-wave to peak mitral annulus velocity ratio, *V̇O*_*2peak*_ peak oxygen consumption.

## Discussion

The present study examined the associations between resting echocardiographic LV structure and function parameters and CRF, as well as exploring the associations between LVM and DXA-obtained body composition (i.e., FFM and FM) in individuals with cervical and upper-thoracic SCI. The results of this study show that none of the resting LV parameters (i.e., structure and function) are associated with V̇O_2peak_ (relative to body mass or FFM). There is a medium association between CRF test-obtained peak O_2pulse_, a surrogate of exercise SV, and resting echocardiographic-obtained SV. Although LV parameters have been shown to be altered following SCI compared to non-injured individuals [7], these resting parameters may not necessarily correlate with reduced CRF levels. Our results show that LVM was positively associated with FFM in individuals with motor-complete (i.e., AIS A and B) and above mid-thoracic level (≥T6) SCI.

Two large cross-sectional studies that recruited individuals referred for an echocardiogram, with no overt cardiac disease, showed that CRF measured in metabolic equivalents was strongly and inversely associated with LV diastolic dysfunction [13, 14]. No associations were observed with systolic function. Likewise, the CARDIA study [15] comprising healthy, middle-age individuals found strong associations between CRF (i.e., treadmill time as a surrogate) and LV diastolic and systolic functions. However, the associations between CRF and diastolic parameters were higher compared with parameters of systolic function. In clinical populations, Franciosa et al [16]. conducted a study on individuals with congestive heart failure and showed that there were no associations between resting LV systolic function (i.e., Q̇, SV, and ejection fraction) and exercise duration. With the same clinical population, Szlachcic et al [17]. also found no association between V̇O_2max_ and cardiac function at rest, except for an inverse association with LV filling pressure (pulmonary capillary wedge pressure as a surrogate).

To the best of our knowledge, Maggioni et al. [30] was the only group that investigated this association between V̇O_2peak_ and resting LV parameters in the SCI population. In this study, 17 males with paraplegia (i.e., T1–L3) were compared against non-injured individuals. In individuals with SCI, and independent of training status, they found that relative V̇O_2peak_ was positively associated (*r* = 0.36 and *P* < 0.05) with aortic flow velocity (a surrogate of SV). This association was documented when trained and untrained participants were combined and was reflected by a higher relative V̇O_2peak_ compared to our cohort (~17.6 mL/kg/min vs. 11.2 mL/kg/min). We used O_2pulse_, a surrogate of exercise SV, and this demonstrated a medium-sized association with the resting echocardiographic-obtained SV. Participants in our study were motor-complete and had a higher NLI (C4–T6). The disparity in V̇O_2peak_ between our results and theirs may be related the inclusion of different cohorts characterized by AIS and NLI or the different modalities used for peak exercise testing (wheelchair ergometer vs. arm ergometer). Besides the participants in the Maggioni et al. study having a greater degree of sympathetic innervation to splanchnic beds, wheelchair propulsion may increase venous return and eventually improve SV. This could be due to the movement mechanics possibly mimicking an abdominal binder [31]. Further discrepancies stem from the difference in echocardiography-related methodologies between the two studies (i.e., M-mode vs. the modified Simpson’s biplane method).

The lack of associations between resting LV parameters and peak CRF in this study is perhaps, unsurprising. During exercise, there are marked changes resulting from the interactions between different body systems, and the interplay of all of these systems cannot be explained simply by their resting state. The sympathetic arm of the autonomic nervous system plays a crucial role in bodily system adjustment in response to physiological stimuli (e.g., exercise, postural challenges). In non-injured individuals, the sympathetically induced vasoconstriction of peripheral vascular beds (splanchnic circulatory bed and lower limbs) and the action of skeletal muscle pumps serve to achieve appropriate venous return and increased end-diastolic ventricular volume (pre-load). As a result, in accordance with the Frank–Starling mechanism, SV is maintained. It might be interesting to speculate that these physiological responses apply to individuals with SCI. However, individuals with injuries ≥T6 experience drastic alterations to these normal physiological mechanisms, along with potentially reduced circulating catecholamines [9], which impact cardiac mechanics and exercise performance. Moreover, the reduced hemodynamic load induced by upper-extremity exercise alone may hinder these mechanisms. In the same context, the lower HR that is frequently observed due to sympathetic decentralization [9] might also result in reduced exercise-related stimuli. Both oxygen transport and extraction mechanisms are affected by the NLI and severity of injury [32]. It should be considered that the aforementioned central exercise-induced mechanisms do not account for the oxygen extraction (peripheral, i.e., skeletal muscle oxidative capacity) that might also be responsible for reduced CRF in individuals with SCI [33].

The cardiovascular system has mainly evolved for the efficient distribution of food/nutrients (e.g., the acquisition and distribution of oxygen) to metabolically active tissues, such as muscles, during an exercise stressor rather than to less metabolically active tissue (i.e., adipose) [22]. Compared to other body composition parameters, FFM represents the body compartments that dominate metabolic demands [34]. Previous studies in non-athletic and athletic adults have shown that FFM is associated with LVM and explains up to 30–37% of the variance in LVM [19, 20]. Our data are in accord with these findings and suggest that among cervical and upper-thoracic SCI, FFM is more strongly associated with LVM than FM, explaining 38% of the variance. Theoretically, this might be useful in the indexing process, as it will allow for distinguishing between metabolically active tissues and those that are less metabolically active.

## Limitations

This study is limited by its cross-sectional design. Thus, further studies are needed to investigate longitudinal changes in CRF and LV parameters. Exercise itself produces marked changes in LV performance; thus, future studies may want to investigate the association between exercise parameters and stress echocardiographic-obtained LV dynamic measures, preferably with the position in which the exercise was performed (i.e., upright or supine). DXA, among other techniques, is the current “gold standard” in determining body composition; however, it is less available in daily clinical practice. Devices such as a bioelectrical impedance analysis may offer a portable and alternative way of measuring body composition [22]. Due to the relatively small sample size, we were unable to perform a multivariate linear regression. Furthermore, the homogeneity and lower CRF levels of the included cohort (i.e., less variability) may impede our ability to detect large or significant associations [35]. Hence, larger studies with more heterogeneous samples are needed to draw more definitive conclusions.

## Conclusion

In conclusion, resting LV parameters in individuals with cervical and upper-thoracic SCI are not associated with peak CRF. Nevertheless, assessing CRF is crucial to quantify the responses to exercise training, irrespective of LV parameters and likely provides an indication of peripheral adaptations and functional improvements. The nature of the associations between LVM and FFM needs further studies to investigate the possibility of using this measure to index LV parameters in the SCI population. Likewise, the association between parameters of CRF and body dimensions needs further investigation to determine the appropriate indexing approaches relative to active musculature (i.e., upper-body FFM).

## Data Availability

The data sets that were collected and analyzed for the purpose of this study are available from the corresponding author upon a reasonable request.
